# Food consumption patterns and associated factors among Vietnamese women of reproductive age

**DOI:** 10.1186/1475-2891-12-126

**Published:** 2013-09-12

**Authors:** Phuong H Nguyen, Garrett Strizich, Alyssa Lowe, Hieu Nguyen, Hoa Pham, Truong V Truong, Son Nguyen, Reynaldo Martorell, Usha Ramakrishnan

**Affiliations:** 1 Thai Nguyen University of Pharmacy and Medicine, Thai Nguyen, Vietnam; 2 International Food Policy Research Institute, Hanoi, Vietnam; 3 Mailman School of Public Health, Columbia University, New York, NY, USA; 4 Hubert Department of Global Health, Rollins School of Public Health, Emory University, Atlanta, GA, USA

**Keywords:** Food frequency questionnaire, Food consumption, Macronutrient intakes, Thai Nguyen, Vietnam, Women of reproductive age

## Abstract

**Background and objectives:**

Adequate nutrient intakes among women of reproductive age (WRA) are important determinants of maternal, neonatal and child health outcomes. However, data on dietary intake for WRA in Vietnam are lacking. This paper aimed to examine the adequacy and determinants of energy and macronutrient intakes among WRA enrolled in a study of preconceptual micronutrient supplementation (PRECONCEPT) being conducted in 20 rural communes in Thai Nguyen province, Vietnam.

**Methods:**

Dietary intakes were determined for 4983 WRA who participated in the baseline survey using a previously validated 107-item (semi-quantitative) food-frequency questionnaire that was administered by trained field workers. Multivariate linear and logistic regression analyses were used to examine factors associated with energy and macronutrient intakes.

**Results:**

A disproportionate number of energy came from starches, primarily rice. Carbohydrate, fat and protein constituted 65.6%, 19.5% and 14.8% of total energy, respectively. Fat intake was below recommended levels in 56.5% of respondents, but carbohydrate intakes were above recommended level in 54.6%. Only 0.1% and 5.2% of WRA achieved adequate intake of n-3 and n-6 long-chain polyunsaturated fatty acids, respectively. Multivariate linear regression revealed that low education, low socioeconomic status, and food insecurity were significant predictors of reduced total energy intake, reduced energy from protein and fat, and greater energy from carbohydrates. Logistic regression confirmed that inadequate macronutrient intake was more common among the poor, food insecure, and less educated.

**Conclusions:**

Imbalanced dietary intakes among underprivileged women reflect lack of dietary diversity. Nutrition programs should be linked with social development, poverty reduction, education programs and behavior change counseling in order to improve the nutritional status of WRA in Vietnam.

## Background

Since the early 1990s, Vietnam has shown substantial reductions in rates of undernutrition [1-4]. The prevalence of chronic energy deficiency (Body mass index - BMI < 18.5 kg/m^2^) among women of reproductive age (WRA) decreased dramatically between 1990 and 2000 (from 48% to 33.1%) [2], and according to a General Nutrition Survey, dropped to 18% in 2010 [5]. While the trend was modest and largely isolated to rich households in the 1990s, there were dramatic increases in total energy intake and improvements in dietary diversity around the turn of the 21st century [3,5]. These and other nutritional changes are likely due to significant economic growth as the result of the implementation of *Doi Moi* economic reforms [6], increased living standards [2], reduced infectious disease morbidity and improvements in dietary consumption patterns [7]. The last two decades have been marked by an overall increase in energy intake, with a shift toward greater consumption of higher quality protein from animal sources and less energy from rice, which has traditionally dominated the Vietnamese diet.

Despite significant improvements over the last decade, chronic energy deficiency is still a significant public health concern, affecting one in every five WRA in Vietnam [5]. More than two thirds of energy comes from starchy staples [3,4], reflecting a high reliance on a few foods and consequently insufficient micronutrient intake in resource-poor settings [8]. Inadequate nutrition intake among WRA carries important implications for not only women’s health, including undernutrition and chronic disease, but also negatively influences pregnancy and child outcomes, including increased risk of neural tube defects, low birth weight, and preterm delivery [9].

There are limited data on dietary intakes of WRA in Vietnam. Several studies at the household level have been conducted including the Vietnam Living Standard Surveys in 1992–1993 [3,10] and 1997–1998 [1,3,10], and the Vietnam Household Living Standard Surveys in 2004 [3] and 2006 [4]. However, these surveys relied on food expenditure to compute intakes. The National Institute of Nutrition (NIN) surveys have been conducted every 10 years since 1990, providing information on national nutritional status and household level macronutrient consumption; however, data in these surveys were collected at the household rather than at the individual level, and none were specific to WRA. Evidence suggests pro-male and pro-adult biases in food intake in South and Southeast Asia [11].

This paper complements previous surveys by analyzing individual level energy and macronutrient consumption among WRA in Thai Nguyen province; it also describes consumption patterns with respect to ethnicity and other demographic and socioeconomic variables.

## Materials and methods

### Data source and study population

Data were obtained from a baseline survey conducted in Thai Nguyen province, in the mountainous region of Northeast Vietnam, as part of a randomized controlled trial (PRECONCEPT study) aimed at improving maternal and infant iron status and overall health [12]. Study subjects were identified from 20 communes located in 4 of Thai Nguyen’s 9 districts with primarily agricultural economies. Women intending to get pregnant within a year were invited to participate in the study. A total of 5011 women of reproductive age (WRA) were included in the baseline survey of which 4983 provided completed demographic and food intake data and were included in this analysis. The study received ethical approval from the Vietnam Institute of Social and Medical Studies and Emory University Institutional Review Boards (IRB).

### Measures

#### ***Dependent variables: food intakes***

The key outcomes in this analysis were total energy and macronutrient intakes (protein, fat and carbohydrate), as well as the percentage of energy from these sources. Dietary intake information was collected using a semi-quantitative food frequency questionnaire (FFQ) developed and validated by NIN [13]. The FFQ includes a list of 107 common food and beverage items that are consumed in North Vietnam. Participants were asked to recall the frequency of consumption of each food item over the last three months. The average portion size for each type of food was determined using commonly used utensils as a reference. Total energy and macronutrient intakes were computed using the Vietnamese food composition tables and aggregating the contribution from all foods [14]. The nutrient content of complex foods that were not included in the database was calculated by identifying the component ingredients from a common Vietnamese recipe book [15]. The 107 food items were also grouped into 10 categories: 1) grains, roots, and tubers; 2) legumes and nuts; 3) vegetables; 4) fruits; 5) oil, lard and butter; 6) meat, offal, and meat products; 7) fish and shellfish; 8) eggs; 9) milk and other dairy products; and 10) sugar, sweets, condiments, and beverages. Energy and macronutrient intakes from each food group were calculated.

The percent energy from macronutrient intakes were compared with the Acceptable Macronutrient Distribution Ranges (AMDR) recommended by US Institute of Medicine (IOM) for assessing insufficient or excessive intake [16,17]. The AMDR is the percent of energy intake that is associated with reduced risk of chronic disease yet provides adequate amounts of essential nutrients. Consuming below or above these ranges implies increased risk of deficiency or chronic disease. For adults, the AMDR for fat, carbohydrate and protein are 20–35, 45–65 and 10-35% respectively, and the AMDRs for n-3 and n-6 fatty acids are 0.6–1.2% and 5–10%, respectively. Estimated energy requirement (EER) [17] was calculated for each individual using the formula EER = 354 - (6.91 × age[y]) + PA × [(9.36 × weight[kg]) + (726 × height[m])], where PA (physical activity coefficient) = 1.27 for farmers and 1.12 for other occupations. The association between actual and predicted intakes was assessed, and a variable was generated for each woman coding whether the actual intake was below the predicted one (1 = yes, 0 = no).

#### ***Independent variables***

Independent variables were considered at the individual and household level. At the individual level, we examined age, education (4 categories based on highest grade level completed), ethnicity (Kinh majority vs. all minority groups) and occupation (farmer vs. other). At the household level, we included socio-economic status (SES), and food security. The SES index was constructed using principal components analysis (PCA) that included house and land ownership, housing quality (e.g., house construction materials), access to services (water, electricity, gas and sanitation services) and household assets (various durable goods, agricultural machinery, animals and livestock) [18,19]. The first component derived from the PCA was then used to categorize SES status into quintiles. Household food security was assessed using Household Food Insecurity Access Scale (HFIAS) developed by Food and Nutrition Technical Assistance Project [20]. The HFAIS is based on behavior and perceptions related to household food insecurity, anxiety and uncertainty, insufficient quality of intake, and insufficient food intake and its consequences. Each participant was given a food security rating of secure, mildly insecure, moderately and/or severely insecure.

### Statistical analyses

All data analyses were performed using SAS version 9.3 [21]. A p-value <0.05 was considered statistically significant. Data were checked for normal distribution using the Kolmogorow-Smirnov test of normality. Observations greater than three standard deviations from the mean for total energy, carbohydrate, fat or protein consumption were considered outliers and removed from the analysis. Overall consumption of energy and macronutrients as well as percent energy from carbohydrate, fat or protein were normally distributed. However, contributions of energy and macronutrients from specific food group category were not normally distributed; thus median and inter-quartile ranges (IQRs) were reported in these cases.

Descriptive analyses were used to report demographic and socioeconomic characteristics of the study sample. Bivariate associations between socio-demographic variables and energy and macronutrient intakes were examined using students T-test and Analysis of Variance (ANOVA). Multivariate linear regression was then applied to examine the main factors associated with total energy intake, and percent energy from carbohydrate, fat and protein. Logistic regression was also used to identify predictors of low energy intake (less than EER) and fat intake (below AMDR). The percentage of insufficient protein intake was very low (0.5%); therefore this variable was not examined in the model. Instead, we modeled the excessive intake of carbohydrate (above AMDR). All models were adjusted for clustering effects at the commune level.

## Results

### Descriptive characteristics

Background characteristics of the study sample were presented in Table 1. Mean age was 26, ranging from 16 to 44 y. Farmers represented 80.6% of the sample, and 50.5% of participants were of the Kinh majority ethnic group. Ethnic minority groups with significant representation in the sample were Tay (17.4%), Nung (12.7%), San Chi (7.0%), Dao (5.4%), and San Diu (2.2%). More than half of participants (54.7%) had completed some or all secondary school; 25.1% completed at least some high school; and 12.0% completed at least one year of higher education, with only 8.2% having less than a primary school education. In terms of household food security, 68.7% women were considered living in a food secure household, whereas 12.4% were categorized as mildly food insecure and 18.9% as moderately/severely food insecure.

**Table 1:** Background characteristics of study participants

	**n**	**%**
**Participant characteristics**		
***Age of woman (mean ± SD: 26.15 ± 4.56)***		
≤ 25	2419	48.57
26-30	1712	34.38
> 30	849	17.05
***Ethnicity***		
Kinh	2515	50.49
Minority	2466	49.51
***Occupation***		
Other	966	19.39
Farmer	4017	80.61
***Education (Highest grade completed)***		
0-5	410	8.23
6-9	2726	54.71
10-12	1250	25.09
Greater than 12	597	11.98
***BMI****(mean ± SD: 19.5 ± 1.9)*		
Underweight (BMI < 18.5 kg/m)	1580	31.71
Overweight (BMI > 23 kg/m)	249	5.00
**Household characteristics**		
***Food Insecurity Level***		
Moderate or severe	942	18.9
Mild	616	12.36
Secure	3425	68.73
***Socioeconomic status***		
Poorest	995	20.01
Poorer	995	20.01
Average	994	19.99
Richer	995	20.01
Richest	994	19.99
***Number of dependents***		
0	386	7.75
1	3697	74.19
2 or more	900	18.06

### Energy and macronutrient intake and food sources

Energy and macronutrient intakes (overall and by food group) are shown in Table 2. Mean measured total energy intake was 2196 kcal/day with 65.5%, 14.8%, and 19.5% coming from carbohydrate, protein and fat, respectively. The main food source of energy was rice and other starchy staples (62.6%), followed by meat and meat products (9.2%) and legumes/nuts/seeds and fruits (5% each). Animal source foods (meat, fish, eggs and dairy) excluding lard constituted 12.7% of total energy intake. The daily intakes of protein, fats and carbohydrates were 82 g, 49 g, and 356 g, respectively. Rice and other staples were the primary source of protein (30 g/day), followed by meat and meat products (17.7 g/day), and pulses, nuts and seeds (7.9 g/day). The main sources of dietary fat intake were meat and meat products (14.0 g/day); oils, lard, and butter (8.6 g/day); and pulses, nuts, and seeds (6.1 g/day). The mean daily fat breakdown was: total saturated fatty acid, 10.7 ± 5.6 g; total monounsaturated fatty acid, 15.8 ± 9.0 g; total long chain polyunsaturated fatty acid (LCPUFA), 7.7 g ± 4.5 g; n-3 LCPUFA, 0.5 g ± 0.2 g; and n-6 LCPUFA, 6.9 g ± 4.3 g (results not shown). Mean total cholesterol intake was 261.2 ± 176.8 mg. Adequate intake of n-3 and n-6 fatty acids as defined by the IOM [17] was achieved by 0.1% and 5.2% of participants, respectively.

**Table 2:** Energy and macronutrient consumption from food groups

	**Energy (% of total)**	**Energy (Kcal)**	**Carbohydrate (g)**	**Fat (g)**	**Protein (g)**
	**Median (IQR range)**	**Median (IQR range)**	**Median (IQR range)**	**Median (IQR range)**	**Median (IQR range)**
Cereals and starchy vegetables	62.6% (54.4% - 70.5%)	1317 (1079–1563)	289.9 (237.2 - 343.3)	4.0 (3.2 - 5.1)	30.0 (24.5 - 35.4)
Pulses, nuts, and seeds	5.0% (2.8% - 8.3%)	105 (52–196)	3.9 (1.8 - 7.3)	6.1 (2.7 - 12.3)	7.9 (4.3 - 13.6)
Vegetables	2.7% (1.8% - 3.7%)	56 (36–86)	8.2 (5.1 - 12.6)	0.2 (0.1 - 0.3)	5.4 (3.2 - 8.3)
Fruits	5.0% (2.9% - 7.6%)	107 (56–179)	23.8 (12.4 - 39.8)	0.1 (0.0 - 0.2)	2.6 (1.4 - 4.3)
Oils, lard, and butter	3.8% (2.3% - 6.2%)	78 (49–132)	0.0 (0.0 - 0.0)	8.6 (5.4 - 14.7)	0.0 (0.0 - 0.0)
Meat and meat products	9.2% (6.3% - 12.7%)	198 (121–297)	0.0 (0.0 - 0.1)	14.0 (8.5 - 21.3)	17.7 (10.8 - 26.6)
Fish and shellfish	0.6% (0.3% - 1.2%)	13 (5–26)	0.0 (0.0 - 0.3)	0.3 (0.1 - 0.6)	2.4 (1.0 - 4.9)
Eggs	2.3% (1.2% - 3.7%)	50 (23–83)	0.2 (0.1 - 0.6)	3.5 (1.7 - 6.0)	3.9 (2.0 - 6.8)
Milk and dairy	0.3% (0.0% - 1.4%)	6 (0–34)	0.3 (0.0 - 2.1)	0.3 (0.0 - 2.1)	0.3 (0.0 - 1.9)
Sugar, sweets, condiments, and beverages	2.4% (1.2% - 4.1%)	50 (23–96)	9.9 (4.0 - 20.0)	0.0 (0.0 - 0.3)	1.6 (0.9 - 2.6)
***Overall (median & IQR)***		2107 (1739–2579)	345.6 (288.4 - 415.3)	44.8 (32.0 - 61.9)	78.3 (61.1 - 99.1)
***Overall (mean & SD)***		2196 ± 650	356.1 ± 98.3	48.7 ± 22.9	82.0 ± 29.0
***Estimated Energy Requirement***^**a**^	NA	1810 ± 97	NA	NA	NA
**Percent insufficient intake**^**b**^			**0.5**	**56.5**	**0.5**
**Percent in optimal range**			**44.9**	**42.8**	**99.5**
**Percent excessive intake**			**54.6**	**0.7**	**0**

^a^Estimated energy requirement (EER) for women was calculated based on the formula suggested by IOM [17]: EER = 354 - (6.91 × age[y]) + PA × [(9.36 × weight[kg]) + (726 × height[m])], where PA (physical activity coefficient) = 1.27 for farmers and 1.12 for other occupations.

^b^Insufficient and excessive intake levels were defined based on the following Acceptable Macronutrient Distribution Ranges (AMDRs): protein, 10-35%; fat, 20-35%, carbohydrate, 45-65% of total energy.

Measured energy intakes were weakly associated with EER (n = 4970, r = 0.04, p = 0.002). Mean measured energy intakes exceeded the mean EER by 386 Kcal (2196 vs. 1810) and about two thirds of the participants had intake values above the calculated EER. Over half (56.5%) did not meet fat intake requirements and 0.7% were classified as having excessive fat intakes based on AMDRs. Almost all study participants exceeded the lower bound of the AMDRs and more than half (54.6%) exceeded the upper bound of the AMDRs for carbohydrate. For protein, although all women had intakes that were within the lower and upper bounds of the AMDRs, only 34.6% of protein came from animal sources (results not shown).

### Bivariate analyses of associations between energy and macronutrient intakes with demographic and socioeconomic factors

Results of the bivariate analyses examining the association between food consumption patterns and maternal and household factors are presented in Table 3. Total energy as well as percent of energy from carbohydrate, fat and protein varied significantly (p-value < 0.05) by participants’ occupation and education, household food security and SES. Women of higher SES, more years of schooling, living in households with greater food security, and non-farmers consumed more total energy and more% energy from fats and protein, but less% energy from carbohydrate compared to their counterparts. Sources of energy intake also varied by ethnicity, with the Kinh ethnic majority consuming more energy from fat and less energy from carbohydrate compared to ethnic minority groups.

**Table 3:** Energy and macronutrient intakes by demographic and socioeconomic status

	**Total energy (Kcal)**			**% Energy from carbohydrate**		**% Energy from fat**		**% Energy from protein**	
	**n**	**Mean ± SD**	**p-value**^**a**^	**Mean ± SD**	**p- value**	**Mean ± SD**	**p- value**	**Mean ± SD**	**p- value**
***Overall***	4920	2196 ± 650		65.5 ± 7.2		19.5 ± 5.6		14.8 ± 2.1	
**Participant characteristics**									
***Age of woman***									
<=25	2393	2214 ± 658	0.03	65.8 ± 7.2	0.02	19.2 ± 5.6	0.01	14.8 ± 2.1	0.25
26-30	1688	2196 ± 644		65.2 ± 7.1		19.7 ± 5.5		14.8 ± 2.0	
>30	836	2146 ± 637		65.3 ± 7.6		19.6 ± 5.9		14.7 ± 2.1	
***Ethnicity***									
Kinh	2489	2184 ± 648	0.17	65.2 ± 7.1	0.002	19.7 ± 5.5	0.001	14.8 ± 2.0	0.38
Minority	2429	2209 ± 652		65.8 ± 7.4		19.2 ± 5.7		14.8 ± 2.1	
***Occupation***									
Other	952	2252 ± 662	0.003	62.0 ± 6.7	<0.001	21.8 ± 5.2	<0.001	15.9 ± 2.0	<0.001
Farmer	3968	2183 ± 646		66.4 ± 7.1		18.9 ± 5.6		14.5 ± 2.0	
***Education (Highest grade completed)***									
0-5	404	2085 ± 658	0.001	68.4 ± 8.0	<0.001	17.3 ± 6.1	<0.001	14.0 ± 2.3	<0.001
6-9	2693	2194 ± 647		66.1 ± 7.0		19.1 ± 5.5		14.6 ± 2.0	
10-12	1234	2214 ± 646		65.0 ± 7.0		19.8 ± 5.4		15.0 ± 2.1	
College or higher	589	2248 ± 658		62.1 ± 6.9		21.7 ± 5.3		15.8 ± 2.1	
**Household characteristics**									
***Food insecurity status***									
Moderate or severe	927	2064 ± 639	<0.001	67.9 ± 7.2	<0.001	17.7 ± 5.8	<0.001	14.2 ± 2.1	<0.001
Mild	611	2119 ± 624		66.4 ± 6.9		19.1 ± 5.6		14.4 ± 2.0	
Secure	3382	2246 ± 651		64.7 ± 7.1		20.0 ± 5.5		15.0 ± 2.0	
***Socioeconomic status quintile***									
Poorest	982	2098 ± 668	<0.001	67.8 ± 7.6	<0.001	17.8 ± 5.9	<0.001	14.0 ± 2.1	<0.001
Poorer	985	2157 ± 620		66.9 ± 6.9		18.6 ± 5.5		14.4 ± 1.9	
Middle	982	2168 ± 623		66.1 ± 6.7		19.1 ± 5.3		14.6 ± 1.9	
Richer	979	2223 ± 632		64.5 ± 6.9		20.1 ± 5.4		15.1 ± 1.9	
Richest	982	2341 ± 677		62.3 ± 6.7		21.6 ± 5.2		15.8 ± 2.0	

^a^P-values are based on student T-test and ANOVA test.

### Multivariate linear regression of factors associated with energy and macronutrient intakes

Multivariate regression analyses revealed that household food insecurity and SES were strongly associated with total energy intake (Table 4). Those classified as “food secure” consumed on average 151 kcal/day more than those classified as moderately or severely food insecure, after adjusting for other factors. When using the poorest quintile as the reference category, those in the richest quintile consumed on average 271 (95% CI: 197, 345) more kilocalories per day. Compared to women with the lowest level of education (primary school), women who had completed middle and high school consumed more energy (78 and 40 kcal respectively). Minorities also consumed on average 43 more kcal/day more than members of the Kinh majority group but there were no differences in the sources of energy. Analyses using linear models comparing the six ethnic groups revealed that the differences are due to significantly higher total energy, protein, and fat consumption among WRA from the San Chi ethnic group (results not shown). Adjusted mean energy intake was 2,255 among San Chi participants, compared to 2,069 kcal/day for women of other ethnic backgrounds (results not shown).

**Table 4:** Socioeconomic and demographic determinants of energy and macronutrient intakes

	**Total energy intake (Kcal/day)**			**Percent energy from carbohydrate (%)**			**Percent energy from fat (%)**			**Percent energy from protein (%)**			**Percent protein from animal sources (%)**		
	**β**	**95% CI**	**p-value**	**β**	**95% CI**	**p-value**	**β**	**95% CI**	**p-value**	**β**	**95% CI**	**p-value**	**β**	**95% CI**	**p-value**
**Participant characteristics**															
***Age***	-2	(-6, 3)	0.47	-0.1	(-0.1, 0.0)	0.001	0.1	(0.0, 0.1)	<0.001	0.00	(0.0, 0.0)	0.29	-0.1	(-0.2, 0.0)	0.001
***Ethnicity (minority vs other)***	43	(4, 81)	0.03	-0.1	(-0.5, 0.3)	0.63	0.0	(-0.3, 0.3)	0.95	0.2	(0.0, 0.3)	0.006	0.6	(0.0, 1.2)	0.05
***Occupation (farmer vs other)***	35	(-31, 100)	0.30	2.1	(1.4, 2.8)	<0.001	-1.4	(-2.0, -0.9)	<0.001	-0.7	(-0.9, -0.5)	<0.001	-4.6	(-5.7, -3.5)	<0.001
***Education (Highest grade completed; ref = 0-5)***															
6-9	78	(9, 147)	0.03	-1.7	(-2.4, -0.9)	<0.001	1.4	(0.8, 1.9)	<0.001	0.4	(0.2, 0.6)	<0.001	2.9	(1.8, 4.1)	<0.001
10-12	43	(-34, 120)	0.27	-1.9	(-2.7, -1.0)	<0.001	1.4	(0.8, 2.1)	<0.001	0.5	(0.3, 0.8)	<0.001	3.7	(2.5, 5.0)	<0.001
College or higher	39	(-60, 138)	0.44	-2.1	(-3.2, -1.1)	<0.001	1.5	(0.6, 2.3)	<0.001	0.5	(0.2, 0.8)	0.007	3.2	(1.5, 4.8)	<0.001
**Household characteristics**															
***Food Insecurity status (ref = moderate or severe food insecurity)***															
Mild	51	(-14, 117)	0.13	-1.1	(-1.8, -0.4)	0.001	1.1	(0.5, 1.6)	<0.001	0.1	(-0.1, 0.3)	0.49	1.9	(0.8, 3.0)	<0.001
Secure	151	(100, 201)	<0.001	-1.8	(-2.4, -1.3)	<0.001	1.4	(1.0, 1.8)	<0.001	0.4	(0.2, 0.5)	<0.001	3.6	(2.8, 4.5)	<0.001
***Socioeconomic status quintile (ref = poorest quintile)***															
Poorer	64	(5, 122)	0.03	-0.4	(-1.0, 0.2)	0.20	0.3	(-0.2, 0.8)	0.24	0.2	(0.0, 0.4)	0.03	1.1	(0.2, 2.1)	0.02
Average	80	(18, 143)	0.01	-0.9	(-1.5, -0.2)	0.01	0.6	(0.1, 1.1)	0.02	0.3	(0.2, 0.5)	<0.001	2.1	(1.1, 3.2)	<0.001
Richer	140	(74, 206)	<0.001	-2.1	(-2.8, -1.4)	<0.001	1.3	(0.7, 1.9)	<0.001	0.7	(0.5, 0.9)	<0.001	4.3	(3.2, 5.4)	<0.001
Richest	271	(197, 345)	<0.001	-3.1	(-3.9, -2.3)	<0.001	2.0	(1.4, 2.6)	<0.001	1.1	(0.8, 1.3)	<0.001	6.5	(5.3, 7.7)	<0.001

Household food insecurity and SES, in addition to a woman’s occupation and education were also significantly associated with energy sources. There was a clear pattern of greater energy intake from protein and fat, and less energy from carbohydrate when comparing higher to lower categories of SES, food security and education. Farmers also consumed a higher percent of total energy from carbohydrate (coefficient: 2.1, 95% CI: 1.4, 2.8) and less from protein and fat compared to non-farmers. Animal foods contributed a lower percent of protein intake in farmers compared to other occupations (coefficient: -4.6, 95% CI: -5.7, -3.5). In contrast, women with higher education, higher SES and more food secure households consumed a higher percent of protein from animal foods.

### Factors associated with increased risk of having energy intakes below the EER, excessive carbohydrate and insufficient fat intake

Results of multivariate logistic regression showing the factors associated with having energy intakes lower than the estimated EER, inadequate intake of fat, and excessive intake of carbohydrate are presented in Table 5. Farmers were 1.5 times more likely to consume inadequate fat, but 1.7 times (95% CI: 1.3, 1.9) more likely to consume excessive amounts of carbohydrate than non-farmers. There were no differences by education for reporting energy intakes below the EER, but there were significant differences for insufficient fat intake and of excessive carbohydrate intake. Women who had completed at least one year of higher education had a 45% reduction in inadequate fat intake, and 48% reduction in excessive carbohydrate intake, when compared with those who had completed primary school. Both food security and SES were positively associated with energy intakes lower than the EERs, inadequate intake of fat, and excessive intake of carbohydrate. When compared with women classified as moderately or severely food insecure, those defined as living in a food secure household were 34% less likely to have energy intakes lower than the EER, 27% less likely to have insufficient fat intake, and 36% less likely to have excessive carbohydrate intake. Women in the highest SES quintile had reduced likelihood of consuming less than the EER (50%), inadequate fat intake (51%) and excessive carbohydrate intake (54%).

**Table 5:** Logistic regression analyses of factors associated with energy intakes below the EER, excessive carbohydrate and insufficient fat intakes

	**Energy intake below EER**^**a**^		**Excessive carbohydrate intake**^**b**^		**Insufficient fat intake**^**c**^	
	**OR (95% CI)**	**p-value**	**OR (95% CI)**	**p-value**	**OR (95% CI)**	**p-value**
**Participant characteristics**						
***Age***	NA^d^		0.97 (0.96, 0.99)	<0.001	0.98 (0.96, 0.99)	<0.001
***Ethnicity (minority vs other)***	0.91 (0.80, 1.04)	0.18	1.00 (0.88, 1.13)	0.95	0.94 (0.83, 1.07)	0.33
***Occupation (farmer vs other)***	NA		1.69 (1.36, 2.09)	<0.001	1.53 (1.24, 1.89)	<0.001
***Education (Highest grade completed; ref = 0-5)***						
6-9	0.84 (0.66, 1.06)	0.14	0.63 (0.49, 0.80)	<0.001	0.64 (0.50, 0.82)	<0.001
10-12	0.97 (0.75, 1.25)	0.82	0.52 (0.40, 0.69)	<0.001	0.55 (0.42, 0.72)	<0.001
College or higher	0.95 (0.69, 1.30)	0.75	0.58 (0.41, 0.82)	0.002	0.58 (0.41, 0.81)	0.001
**Household characteristics**						
***Food Insecurity status (ref = moderate/severe)***						
Mild	0.89 (0.72, 1.11)	0.30	0.69 (0.56, 0.87)	0.001	0.74 (0.60, 0.93)	0.008
Secure	0.66 (0.56, 0.78)	<0.001	0.64 (0.54, 0.76)	<0.001	0.73 (0.61, 0.86)	<0.001
***Socioeconomic status quintile (ref = poorest quintile)***						
Poorer	0.76 (0.62, 0.93)	0.007	0.95 (0.78, 1.15)	0.57	0.89 (0.73, 1.09)	0.25
Average	0.85 (0.69, 1.05)	0.12	0.78 (0.63, 0.96)	0.02	0.78 (0.63, 0.96)	0.02
Richer	0.63 (0.50, 0.79)	<0.001	0.58 (0.47, 0.73)	<0.001	0.62 (0.50, 0.77)	<0.001
Richest	0.50 (0.39, 0.65)	<0.001	0.46 (0.36, 0.58)	<0.001	0.49 (0.39, 0.63)	<0.001

^a^Lower actual intakes than the Estimated Energy Requirement (EER).

^b^Greater than 65% of total energy intake.

^c^Less than 20% of total energy intake.

^d^Age and occupation were used to calculate EER; therefore these variables are not included in the model to predicting the odds of having energy intakes below the estimated EER.

## Discussion

Several large, national studies have assessed energy and macronutrient intake patterns at the household level in Vietnam since the 1990s. However, these studies were based on household level data and none focused specifically on WRA. The generalizability of dietary intakes based on household level data is questionable especially for WRA. Previous work has shown differences in intra-household patterns of food consumption and the role of gender bias that do not favor WRA especially in South and South-East Asia [11]. Moreover, studies using data from the Vietnam Household Living Standard Surveys relied on expenditure data to compute intakes, and did not specify how intakes were estimated for foods without information on amounts purchased [4]. Other studies employed aggregate consumption data across food groups to estimate energy and nutrient consumption from Vietnamese [4] or international food composition tables [3,22], and treated foods consumed outside the home as a single category. In contrast, this study presents data obtained at the individual level from WRA using a previously validated semi-quantitative FFQ and a food composition database compiled specifically for use in Vietnam, estimating energy and nutrient intakes from all foods, whether consumed inside or outside the home.

The mean energy intake in our study population was 2,196 kcal/day, similar to estimates from previous studies [1,4,5,10]. We found that the mean energy intake was higher than the mean EER by 386 kcal, but the poor correlation between measured energy intakes and EER values suggests that comparison of means may have little value. Furthermore the estimation of energy requirements using the IOM formula [17] may be problematic since it is based on Caucasians and does not account for possible differences in body composition. It is therefore impossible for us to assess whether the actual intakes are low or excessive, because the biases in these estimates are unknown. However, we were still able to identify significant relationships between reported energy intakes with household food security and SES in the expected direction which is useful for policy makers.

The most recent NIN survey (2010) found that protein, fat, and carbohydrates constituted 14%, 18%, and 68% of total energy in the northern midlands and mountainous region [5] which are similar to our findings of 14.8%, 19.5%, and 65.5%. Although these results suggest improvements in the consumption of animal based foods and reduced consumption of staples compared to previous studies [1,10,23], there remain dietary imbalances mainly derived from a large proportion of total energy intake from carbohydrate. The results also suggest that food consumption patterns are not improving as fast as the country’s economy.

Nearly all our participants were within the optimal range for protein consumption, which is beneficial in maintaining lean muscle mass and reducing the incidence of chronic disease [24]. However, only a third of protein came from animal source foods which have high bioavailability for several micronutrients. The percent of energy coming from animal sources is greater than reported in previous studies [4] and the percentage of energy from rice and other staples has decreased from an estimated 80% in 1998 [10] to 66% in our study, implying an improvement in dietary diversity. However, the majority of participants still consume 66% of their total energy from carbohydrates, particularly from rice and other staples, indicating poor dietary diversity overall. Further improvements in dietary diversity could play an important role in decreasing undernutrition among WRA and improving micronutrient intake [8].

Fat intakes were low and more than half (56.5%) consumed <20% energy from fat which is the lower limit recommended by IOM [17]. Increasing fat intakes could help increase energy intakes especially among food insecure households in developing countries, but ecological and observational data suggests that shifting from a low fat diet to a high fat diet as a percent of total energy is associated with an increase in unhealthy weight gain, and potentially contributes to obesity, diabetes, and other chronic conditions [17]. We were able to examine the type of fat consumed using the Vietnamese food composition tables that were revised in 2007 and have reliable data on the fatty acid composition of foods that were determined using chromatographic methods [14]. Mean saturated fatty acid and cholesterol intakes are within levels recommended by the IOM to prevent cardiovascular and other chronic diseases [17] but the intakes of n-3 and n-6 LCPUFAs are far below the recommended dietary intakes. This is a concern in light of recent evidence demonstrating the benefits of n-3 fatty acids for improving pregnancy outcomes and reducing the risk of cardiovascular disease [25-27]. Therefore, increasing fat intakes, especially n-3 fatty acids, may be beneficial if this is accompanied by reductions in carbohydrates, which is associated with increased risk of diabetes and cardiovascular disease [17].

We used the AMDR values recommended by the IOM to evaluate the adequacy of macronutrient intakes [17] instead of the Vietnamese recommendations [28] because of the lack of values for protein. The suggested ranges for fat are similar in both recommendations but the AMDR for carbohydrates are much higher and narrower in the Vietnamese recommendation (61-70%) compared to IOM (45-65%). The Vietnamese recommendation classifies an estimated 26% of women with deficient carbohydrate intakes, compared to only 0.6% using the IOM and to 2.2% using the WHO recommendations [29]. It seems unlikely that around a quarter of Vietnamese women have deficient carbohydrate intakes. For these reasons we decided to use the ranges recommended by the IOM [17].

Ethnic minorities make up a disproportionately large proportion of the poor in Vietnam and they have significantly lower living standards, including less access to healthcare and education [30]. Childhood malnutrition among rural minority populations did not decrease during the 1990’s as it did among the Kinh majority [10], and minority children under five were nearly twice as likely to be both moderately and severely malnourished in 2006 [4]. Minorities have also been reported to be more likely to be anemic [31]. Nearly 50% of our study population sample identifies itself as coming from an ethnic minority. Contrary to expectations, dietary energy intakes (overall and from protein) were greater among minorities compared to the majority Kinh in our study population, after adjusting for other factors. There were no differences in the amount of energy from fat and carbohydrate or the proportion having low protein and fat intakes. To our knowledge, no other study in Vietnam has examined food consumption pattern among ethnic minorities.

We found that food security and SES were the significantly and positively associated with energy intakes (overall and from protein and fat), and negatively associated with carbohydrate intakes. The odds of having energy intake lower than the EER, and insufficient intake of protein and fats were also higher among those from lower SES and food insecure households. These differences were not only statistically significant, but large and of public health significance. For example, energy intake was lower by 151 kcal among food insecure compared to food secured women and lower by 271 kcal in the lowest quintile compared to the highest quintile of SES. Similarly, insufficient fat and excessive carbohydrate intake were around 30% higher for food insecure compared to food secured women; and 50% higher for the lowest quintile compared to the highest quintile of SES. While increasing SES does not necessarily translate into improved nutritional status in developing countries [32], these findings suggest that overall intake, protein intake, and dietary diversity can be improved by targeting interventions to the poor and food insecure. Higher education was also associated with higher intakes of animal foods, which has been shown in previous studies and can be viewed as a marker of better diet quality in this population [33,34]. We did not find major differences by occupation except that farmers were more likely to have lower fat intakes.

Key strengths of our findings include: 1) the large sample that is fairly representative of the northern mountainous areas of Vietnam, 2) the use of standardized methods that rely on a previously validated semi-quantitative food frequency questionnaire administered by well-trained interviewers, and 3) detailed information on sociodemographic characteristics that include measures of food security, ethnicity, socioeconomic status and education. Our findings can be generalized to the population of WRA in Thai Nguyen province and to the wider region of northern Vietnam with important policy implications for improving women’s health and nutritional status. The availability of detailed information on locally consumed foods with a food composition table designed specifically for the local diet are also important strengths of the study that allowed us to carefully examine the overall and detailed profile of intakes by nutrient and type of food. Nevertheless, there are some important limitations that call for caution in the interpretation of our findings. Most notably, the weak correlation between actual energy intakes and estimated energy requirements is a major concern which limits our ability to make conclusions on the adequacy of energy intakes in this population. This may be due to measurement error and/or the need for better methods to estimate energy requirement in this study population. For example, the use of repeated 24 hour recalls to assess intakes and/or the inclusion of measures of physical activity would have been useful. However, we are confident of our findings related to the patterns and determinants of macronutrient consumption as percentages of energy intake that have important implications for future work in this population.

## Conclusions

WRA with lower education, farmers, and those living in households with lower SES and less food security fall behind their counterparts in high quality food intake and more likely to compensate by carbohydrate intake. Emphasis on replacing some of the rice consumed with high quality protein foods and fats will improve dietary diversity, and adding fish to the diet will help improve intake of n-3 LCPUFA, which is severely lacking in the study population. Poor dietary intakes and diet quality among underprivileged women suggests that nutrition programs and policies should be linked with social development programs such as poverty and inequity reduction, and education improvement, in order to improve the nutritional status of WRA in Vietnam.

## Competing interests

None of the authors had financial or non-financial competing interests.

## Authors’ contributions

PHN participated in writing the proposal, designing the study, oversaw data collection, guided and supervised the statistical analysis, drafted and revised the manuscript. GS performed the statistical analysis and assisted in writing the manuscript. AL assisted in study coordination and in manuscript preparation. HN assisted in study coordination, data collection and manuscript preparation. HP participated in field supervision, data collection and manuscript preparation. TVT participated in data management and manuscript preparation. SN participated in field work organization and manuscript preparation. RM participated in writing the proposal, study design, specification of the analytic approach to assessing diets and drafting the manuscript. UR participated in writing the proposal, study design and drafting the manuscript. All authors contributed in the development, review and approval of the final manuscript.

## Authors’ information

PHN - Phuong H Nguyen, MD, PhD is a a faculty member of the Department of Scientific Research and International Relationship, Thai Nguyen University of Medicine and Pharmacy, Vietnam, and a research fellow at Poverty, Health and Nutrition Division, International Food Policy Research Institute, Vietnam. Tel: +84 0945195395. E-mail: P.H.Nguyen@cgiar.org

GS - Garrette Strizich, BSc is a MHP student at Mailman School of Public Health, Columbia University. Email: gms2171@columbia.edu

AL – Alyssa Lowe is a researcher of the Hubert Department of Global Health, Rollins School of Public Health, Emory University, Atlanta, GA, USA. Email: alyssaelowe@gmail.com

HN - Hieu Nguyen, MSc is a researcher of the Research Unit, Thai Nguyen University of Medicine and Pharmacy, Vietnam. Email: hieunt1234@gmail.com

HP - Hoa Pham, MD, MPH is a faculty member of the Department of Obstetrics and Gynecology, Thai Nguyen University of Medicine and Pharmacy, Vietnam. Email: hoapham461@yahoo.com

TVT - Truong V Truong, MD is a faculty member of Thai Nguyen University of Medicine and Pharmacy, Vietnam. Email : truongviettruong@gmail.com

SN - Son Nguyen, MD, PhD is a faculty member of the Department of Pediatrics, Thai Nguyen University of Medicine and Pharmacy, Vietnam. Email: vansonyk@yahoo.com

RM - Reynaldo Martorell, PhD is a a faculty member of the Hubert Department of Global Health, Rollins School of Public Health, Emory University, Atlanta, GA, USA. Email: rmart77@emory.edu

UR - Usha Ramakrishnan, PhD is the principal investigator of the study and a faculty member of the Hubert Department of Global Health, Rollins School of Public Health, Emory University, Atlanta, GA, USA. Email: uramakr@emory.edu
